# QSAR Models for Repeated Dose Toxicity in Rats Using the CORAL Software

**DOI:** 10.3390/toxics14040338

**Published:** 2026-04-17

**Authors:** Alla P. Toropova, Andrey A. Toropov, Nadia Iovine, Gianluca Selvestrel, Alessandra Roncaglioni, Emilio Benfenati

**Affiliations:** Department of Environmental Health Science, Istituto di Ricerche Farmacologiche Mario Negri IRCCS, Via Mario Negri 2, 20156 Milano, Italy; andrey.toropov@marionegri.it (A.A.T.); nadia.iovine@marionegri.it (N.I.); gianluca.selvestrel@marionegri.it (G.S.); alessandra.roncaglioni@marionegri.it (A.R.); emilio.benfenati@marionegri.it (E.B.)

**Keywords:** NOAEL, QSAR, correlation intensity index, index of ideality of correlation, Las Vegas algorithm, Monte Carlo method

## Abstract

The evaluation of the safety of chemical substances requires the identification of a safe dose, which has no adverse effects on humans. This is obtained through animal studies, with exposure prolonged for months. Repeated-dose toxicity is a term in toxicology and pharmacology referring to the highest tested dose of a substance, so-called No Observed Adverse Effect Level (NOAEL). Experimental data on NOAEL taken from the literature and the OpenFoodTox database (total *n* = 848). To speed up the processing of the enormous number of substances we are exposed to, in silico models are an attractive solution. Monte Carlo technique, incorporating the Las Vegas algorithm, was applied to develop models for repeated-dose toxicity in rats. Optimal descriptors were calculated using correlation weights for attributes of the Simplified Molecular Input Line Entry System (SMILES). Computational experiments were conducted 5 times, with splits obtained using the Las Vegas algorithm. Good predictive potential was observed for these models, with an average determination coefficient on the validation set of 0.77 ± 0.04.

## 1. Introduction

Humans are continuously exposed to chemical substances through diet and environmental sources, and a central objective of chemical risk assessment is to establish protective exposure limits that safeguard public health against long-term effects [1]. Traditionally, experimental repeated dose toxicity (RDT) studies conducted in animal species, most commonly rats, mice, dogs, and rabbits, as described by the U.S. Environmental Protection Agency (USEPA) [2] and the Organization for Economic Cooperation and Development (OECD), have played a key role in establishing safe exposure levels for chemical substances [3,4,5,6]. These studies accounted for 90% of animal use and 70% of chemical toxicity. Experimental determination of the No Observed Adverse Effect Level (NOAEL) is quite complex and expensive. Therefore, assessing this endpoint through computer experiments is an attractive alternative to real-world experiments. There is growing concern about the safety evaluation of chemicals, as their impacts on the environment, animal health, and human health are becoming increasingly evident [7].

The EU’s Natural Substances Strategy for Development [8] has drawn considerable comment from regulators and many scientists for its approach to threats and its intention to mitigate minor, identified risks [9,10]. This is important given the growing dichotomy and different approaches to setting risk dose levels for certain compounds [11] and across industries, as illustrated by recent recommendations from the European Chemicals Agencies [12,13,14].

The NOAEL and the Lowest Observed Adverse Effect Level (LOAEL) are endpoints useful from ecological and medicinal perspectives [15,16,17,18,19,20]. The NOAEL is the highest experimental dose at which no significant adverse response is observed, and the LOAEL is the lowest dose at which adverse effects are observed relative to a control group; indeed, determining the NOAEL of a substance is an important step in safety and regulatory assessments [16,17]. Experimental design, dose selection, exposure duration, target organs, and species responses, leading to considerable variability and uncertainty across studies [17], influence NOAEL outcomes. Despite their regulatory importance, experimentally determining NOAELs for the vast number of existing and newly developed chemicals is neither feasible nor sustainable. Ethical concerns, high costs, long testing times, and limited throughput constrain the applicability of in vivo RDT studies. The European Commission (EC) promotes a transition toward animal-free, new-approach methodologies for chemical safety assessment, exemplified by cosmetics legislation and REACH (Registration, Evaluation, Authorization, and Restriction of Chemicals), while emphasizing the critical role of repeated-dose toxicity evaluation to characterize adverse effects arising from sustained exposure within the regulatory framework [14]. In this context, in silico approaches, such as Quantitative Structure-Activity Relationship (QSAR) models based on chemical structure information, are commonly used to predict chemical toxicity in the absence of experimental data [21,22,23,24,25,26,27,28] and have emerged as promising alternatives for supporting repeated-dose toxicity assessment. Determination of the NOAEL of a substance is an important step in safety and regulatory assessments. Nevertheless, application of conventional in silico strategies, for example, QSAR models, to predict NOAEL values is inherently problematic [22,23,24,25,27] because predicting toxic effects following repeated exposure remains highly challenging due to the complexity of the phenomenon and the multifactorial nature of systemic toxicity [28].

The model by Ghosh and Roy [15] utilizes the q-RASAR v4.1 software. This software requires calculating molecular descriptors and specific sub-models related to the local environment, using a read-across strategy adapted to the substances to be analyzed. It is based on the OpenFoodTox data, as in our study (we used a more recent version of OpenFoodTox), but we also used many more data sources from the literature, as we described. Thus, we can assume that our model has a larger basis and coverage. Indeed, our validation sets comprise more than 200 substances, exceeding the 186 data points used to develop the q-RASAR model, which validated only 27 substances. The statistical parameters of the q-RASAR model are better, likely due to a more sophisticated strategy that incorporates molecular descriptors and more complex read-across algorithms. Indeed, our model is much simpler: it does not require computing molecular descriptors, and the equations we use are those described above. Another attempt to model NOAEL was proposed by Hisaki et al. [24], who developed models using an artificial neural network trained on a dataset of 421 substances and descriptors received from molecular orbital calculations. A tenfold cross-check yielded an RMSE of 0.529 logarithmic units.

The models for NOAEL, built with the OECD QSAR Toolbox of some components of cannabis sativa [25], use physicochemical and logical-structural descriptors (e.g., air-water partition coefficient model, number of single bonds, etc.).

Our study aims to assess the CORAL software by using the Las Vegas algorithm [29] to select a prospective split of available data into training and validation sets (Istituto di Ricerche Farmacologiche Mario Negri IRCCS, available online: http://www.insilico.eu/coral, accessed on 14 April 2026) as a tool to simulate repeated dose toxicity in rats.

## 2. Materials and Methods

### 2.1. Data

Numerical data on 1100 negative decimal logarithms of NOAEL were taken from the literature [15,16,18,19] and from the OpenFoodTox database [20]. The endpoint considered is the negative decimal logarithm of the NOAEL, expressed in mg/kg bw/day. The SMILES of compounds considered were canonized using the VEGAHUB platform, Istituto di Ricerche Farmacologiche Mario Negri IRCCS (available online: https://www.vegahub.eu, accessed on 8 April 2026). After removing duplicates, the total working set consisted of 848 compounds. Experimental data from the literature expressed in terms of negative common logarithms are used as the endpoint for QSAR analysis.

**Figure 1:**
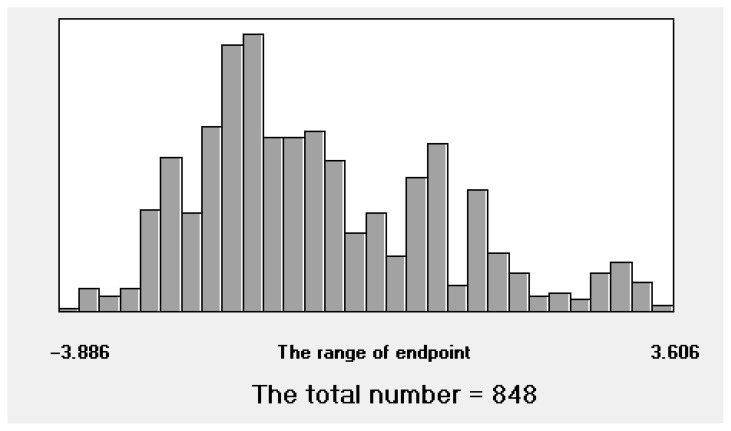
The histogram of experimental values on pNOAEL for all 848 compounds considered.

The histogram of the pNOAEL numerical values for all 848 compounds demonstrates their representativeness (Figure 1), although the region from −1.7 to −1.3 is slightly more populated than the other intervals.

### 2.2. Model

The traditional approach to building QSAR models is to develop a model with good correlation on the training set, hoping that the correlation will hold for the external validation set as well. The correlation balance scheme used here is based on partitioning the training set into three sets: active training, passive training, and calibration. It is more complex than the usual scheme with a single training set. The model is complete when all parameters are optimized, which occurs in the last phase of the modeling process using the calibration set. Thus, the results obtained with the substances in the calibration sets are closer to those usually obtained when a single training set is used to build the model. The populations of the specified sets were chosen to be approximately equal. Table S1 (Supplementary Materials) contains data on the percentage of matching distributions for the five splits considered.

The model for NOAEL is defined as

(1) $$pNOAEL=C_{0}+C_{1}\times DCW(T,N)$$

where T and N are parameters of the Monte Carlo optimization to calculate optimal correlation weights (CWs). T is the threshold that determines whether the SMILES attribute will be included in the model-building system. If a SMILES attribute occurs in the active training sample fewer than T times, it is considered rare and is not considered further (i.e., its correlation weight is set to zero). N is the number of epochs of optimization of correlation weights of SMILES attributes included in the simulation system. One epoch is a cycle of modification of correlation weights for all active (i.e., non-rare) SMILES attributes. C_0_ and C_1_ are regression coefficients, defined by the least squares method.

Computer experiments using the CORAL software showed that the most practical value for the specified optimization parameter T is T = 3 for N = 15.

It is possible that using a nonlinear model could enhance the predictive potential of such models. Perhaps relevant computer experiments will be conducted in the future.

The DCW is the descriptor defined as the sum of the CWs:

(2) $$DCW\left(T,N\right)=\sum CW\left(S_{k}\right)+\sum CW\left({SS}_{k}\right)$$

Four types of SMILES attributes are used for the models considered here. First, SMILES atoms, denoted by S_k_; these attributes are either molecular features represented by a single symbol (e.g., ‘O’, ‘C’, etc.), or a group of symbols (e.g., ‘Cl’, ‘Br’, ‘@@’, etc.) that cannot be considered separately. Second, pairs of SMILES atoms, which follow each other in the SMILES notation. SMILES attributes of this type are denoted by SS_k_.

To ensure that the model is reproducible, one should build it for several different splits of the available data into training and validation sets and verify that the results are similar across splits.

Then, the training set is split further into three parts: (i) active training set, (ii) passive training set, and (iii) calibration set. Supplementary materials (Table S2) show the correlation weights for split 1 (TF_1_). Table 1 shows an example calculation of DCW(3,15) for the SMILES in the dataset under consideration.

Starting from the SMILES, the software identifies specific components based on one or two symbols and uses their coefficients to generate the prediction. The dots near the SMILES symbols are open positions in the software’s format, for inserting additional characters if needed.

### 2.3. Optimization

Equation (2) requires numerical data for the correlation weights. Monte Carlo optimization is a tool for calculating those correlation weights. Here, two target functions for the Monte Carlo optimization are examined:

(3) $${TF}_{0}=r_{A}+r_{P}-\left|r_{A}-r_{P}\right|\times 0.1$$

(4) $${TF}_{1}={TF}_{0}+(IIC+CII)\times 0.5$$

The $r_{A}$ and $r_{P}$ are the correlation coefficients between the observed and predicted endpoint for the active training and passive training sets, respectively.

The index of ideality of correlation (IIC) is calculated as follows [30]:

(5) IICC=rCmin(MAEC−,MAEC+)max(MAEC−,MAEC+)

(6) $$\min\left(x,y\right)=\left\{\begin{array}{c} x,\;if\;x<y\\ y,otherwise \end{array}\right.$$

(7) $$\max\left(x,y\right)=\left\{\begin{array}{c} x,\;if\;x>y\\ y,otherwise \end{array}\right.$$

(8) MAEC−=1N−∑∆k, N− is the number of ∆k<0

(9) MAEC+=1N+∑∆k,N+ is the number of ∆k≥0

(10) $$\Delta_{k}={observed}_{k}-{calculated}_{k}$$

Equation (10) gives the difference between the observed and calculated endpoint values.

The correlation intensity index (CII), similar to the IIC above, was developed to improve the quality of Monte Carlo optimization for building QSPR/QSAR models. Thus, TF_1_ is an improved approach that may yield better results.

The CII is calculated as follows [31]:

(11) $${CII}_{C}=1-\sum {Protest}_{k}$$

(12) $${Protest}_{k}=\left\{\begin{array}{cc} R_{k}^{2}-R^{2}, & if\;R_{k}^{2}-R^{2}>0\\ 0, & otherwise \end{array}\right.$$

The R^2^ is the correlation coefficient for a set that contains n substances. The $R_{k}^{2}$ is the correlation coefficient for n − 1 substances of a set, after removing the k-th substance. Hence, if the ($R_{k}^{2}$ − R^2^) is a larger zero, the k-th substance is an “oppositionist” for the correlation between experimental and predicted values of the set. A small sum of “protests” means a more “intensive” correlation.

**Figure 2:**
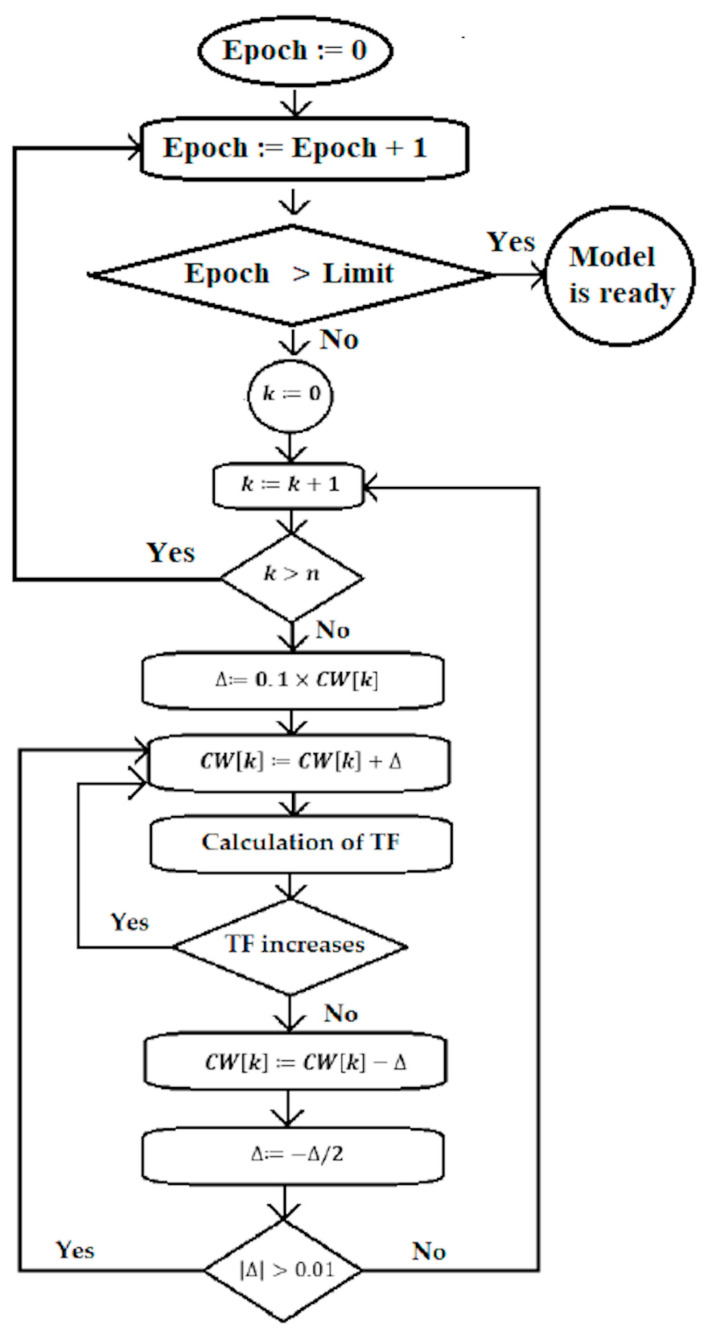
Flowchart of the Monte Carlo optimization.

Figure 2 contains the flowchart of the Monte Carlo optimization.

Each run of the Monte Carlo optimization procedures began with the Las Vegas algorithm applied to the studied data on molecular structure and repeated toxicity dose in rats. The Las Vegas algorithm can be expressed conceptually as: “If I play for as long as I want, I’m bound to win eventually.” In casino practice, such player tactics will make the casino owner rich (though he is already rich, as a rule), but not the players. However, if the goal is to find the most advantageous distribution for the calibration set in the training and validation sets, then ten trials of such distributions are quite feasible. Selecting the best data split in the specified sense for training and validation from 10 attempts is entirely feasible, even for larger datasets.

**Figure 3:**
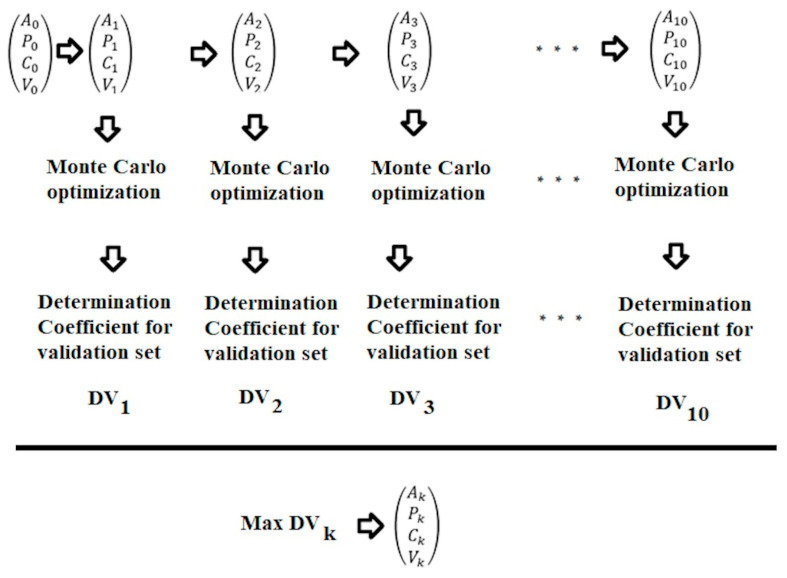
General scheme of the Las Vegas algorithm process.

Figure 3 shows the general scheme of the Las Vegas algorithm. All scatterings are random, but the selection of the distribution of available data in active and passive training sets, as well as calibration and validation sets, surely cannot be examined as random ones.

The Las Vegas algorithm [29] guarantees a result at the expense of time complexity, while Monte Carlo compromises the result guarantee by terminating after executing all instructions of the corresponding program. If a solution is needed, the Las Vegas algorithm will run until it finds the expected solution, whereas Monte Carlo will run for several cycles and stop even if it does not find a solution.

Thus, this study sought to develop a unified framework that integrates the Monte Carlo method and the Las Vegas algorithm. Since the task assigned to the Las Vegas algorithm is to “find the best partition into four special sets, i.e., an active training set, a passive training set, a calibration set, and a validation set,” the Las Vegas algorithm always returns the required “solely one best from ten considered” solution. For greater consistency with general theory, it should be noted that ten is chosen arbitrarily and could easily be replaced by 100 or 1000, thereby confirming the Las Vegas algorithm’s “time complexity” claims.

The advantage of the objective function TF_1_ over TF_0_ lies in reorienting the Monte Carlo process from improving the statistical quality of the model on the training sets to using the training sets’ information to improve the statistical quality of the model on the calibration set. This task is much more complex than improving the statistics on the training samples, but it apparently yields a strange yet useful compromise. The system strives to select the regression coefficients C_0_ and C_1_ so that they fit the calibration set as accurately as possible, sacrificing variance on the training samples. Importantly, this compromise can be beneficial for the statistical quality of the validation set.

**Figure 4:**
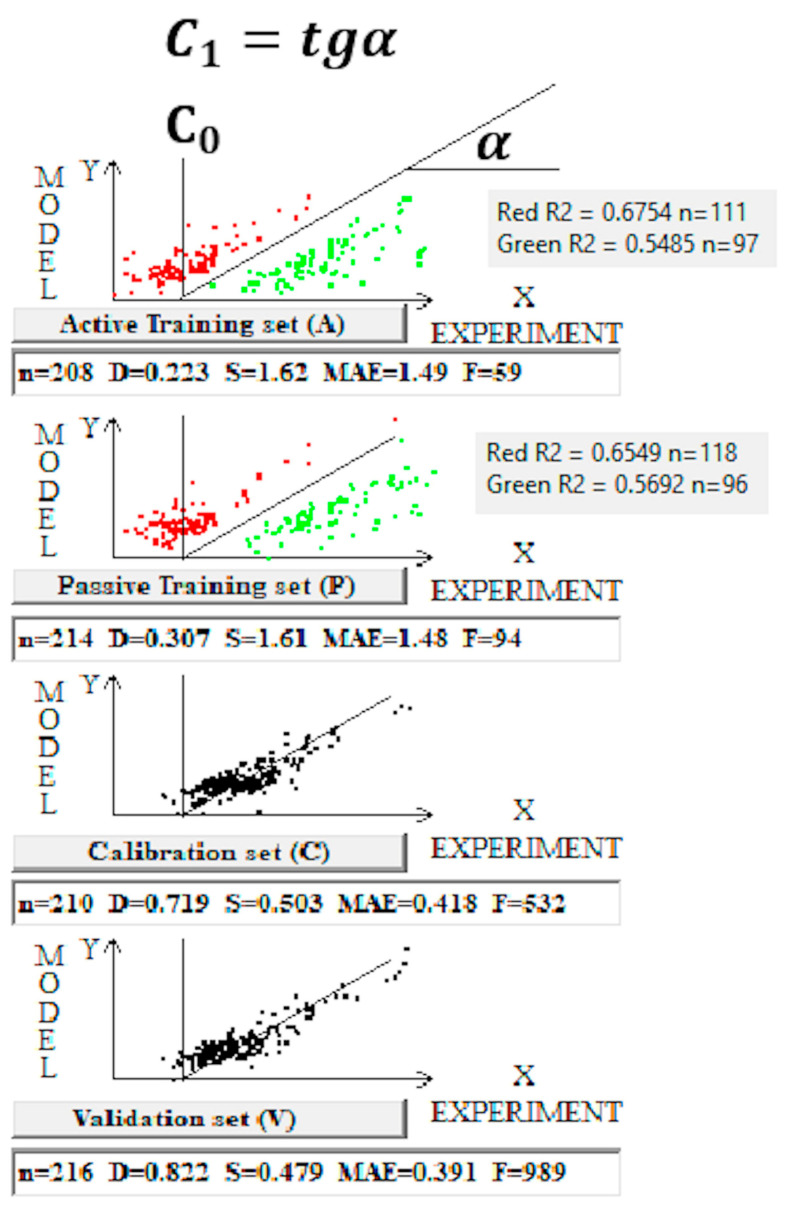
Graphical representation of the Monte Carlo optimization with target function TF_1_ for split 1.

Figure 4 graphically illustrates these assumptions.

Red dots correspond to overestimated toxicity predictions, while green dots represent underestimated predictions. If these red and green dots are considered as a single cluster, the coefficient of determination is quite low. However, the coefficients of determination for the red- and green-dot groups are significantly higher.

### 2.4. Applicability Domain

The applicability domain of the described model is specified by inequality 15, which captures the so-called statistical defects of SMILES attributes. These defects can be calculated as:

(13) $$d_{k}=\frac{\left|{P(A}_{k})-{P^{\prime }(A}_{k})\right|}{N\left(A_{k}\right)+N^{\prime }(A_{k})}+\frac{\left|{P(A}_{k})-{P^{\prime \prime }(A}_{k})\right|}{N\left(A_{k}\right)+N^{\prime \prime }(A_{k})}+\frac{\left|{P^{\prime }(A}_{k})-{P^{\prime \prime }(A}_{k})\right|}{N^{\prime }\left(A_{k}\right)+N^{\prime \prime }(A_{k})}$$

where P(A_k_), P′(A_k_) P″(A_k_) are the probability of A_k_ in the active training set, passive training set, and calibration set, respectively; N(A_k_), N′(A_k_), and N″(A_k_) are frequencies of A_k_ in the active training set, passive training set, and calibration set, respectively. The statistical SMILES-defects (D_j_) are calculated as:

(14) $$D_{j}=\sum_{k=1}^{NA}d_{k}$$

where NA is the number of non-blocked SMILES attributes in the SMILES.

A SMILES falls in the applicability domain if

(15) $$Dj<2*\bar{D}$$

The applicability domain in this definition is more advisory than restrictive. Nevertheless, this definition has enough rationale. The number of potential outliers for the validation set (split 1) according to the approach considered is 20%. The presence of rare molecular features (extracted from SMILES) in the corresponding SMILES is used as a criterion for determining the potential probability of a molecule being an outlier. The usefulness of this approach can be demonstrated by considering a model without the specified outliers (molecules outside the applicability domain).

### 2.5. Mechanistic Interpretation

The mechanistic interpretation [30,31] of the considered models is determined by the stable, either positive or negative, correlation weights of the SMILES attributes observed in a series of Monte Carlo optimization runs. However, the selection of growth promoters or reducers for the endpoint under consideration must be based on the frequencies of occurrence of each candidate increase promoter or decrease in toxicity across the active training set, passive training set, calibration set, and validation set.

## 3. Results

The computational experiments were conducted in two stages. In the first stage, the Las Vegas algorithm [29] was used to find the most favorable distribution for the calibration set. In the second stage, a model was built using this distribution. Table 2 contains the statistical characteristics observed for each of the five splits. The results are reproducible for the five splits considered. The Monte Carlo optimization was performed using the target functions TF_0_ and TF_1_. Full statistics on active and passive training sets are shown in Table S3 (Supplementary Materials). Table S1 presents the overlap percentages for the five splits considered. Table S2 contains SMILES notations together with experimental and calculated pNOAEL values, as well as the correlation weights for SMILES attributes for split 1.

One can see that Monte Carlo optimization with the target function TF_1_ gives models with significantly better statistical quality. In other words, applying the index of ideality of correlation, together with the correlation intensity index, improves the approach’s predictive potential. Furthermore, a significant advantage of TF_1_ models is the greater consistency in the model’s statistical quality across the five considered splits of the data into training and validation sets. Reproducibility of results (lower variance in the determination coefficient values across the validation sets) is an attractive indicator of this approach. A very intriguing point of this study is that the correlation perfection index and the correlation intensity index, for the set of compounds under consideration, when simultaneously involved, improve the statistical characteristics of the model obtained by the Monte Carlo method using the Las Vegas algorithm; however, separately they do not provide good statistical quality of the models under the same conditions (that is, using the Monte Carlo technique and the Las Vegas algorithm).

The average coefficient of determination for the validation set (TF_1_) across the five models is 0.77 ± 0.04.

The model observed for split 1, if the use of the target function TF_1_ is the following:

pNOAEL = −0.9002 (±0.0083) + 0.1524 (±0.0013) × DCW(3,15)(16)

**Figure 5:**
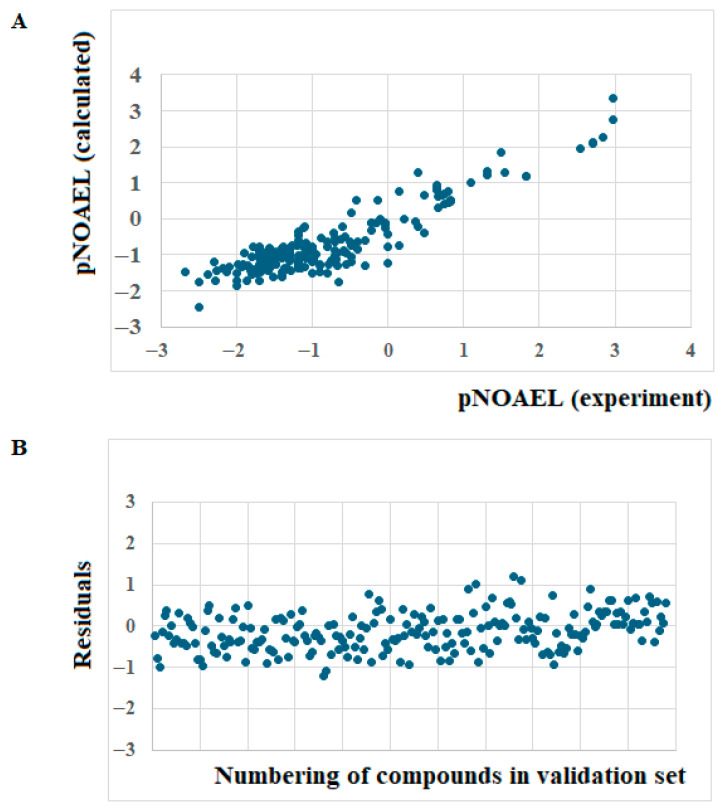
(**A**) Graphical representation of experimental vs. calculated with Equation (16) pNOAEL values for 216 compounds of the validation set (split 1), and (**B**) Standardized residuals plot for the model calculated with Equation (16). Standardized residuals are shown for all 216 compounds of the validation set (split 1) ordered by compound number.

Figure 5 contains the scatter plot and residuals plot for the validation set of the model (Split 1, target function TF_1_).

## 4. Discussion

The methodology for constructing rat toxicity models used in this study differs from traditional approaches to developing models for various endpoints.

First, it involves structuring the training set into three specific subsets: (i) an active training, (ii) a passive training, and (iii) a calibration.

Second, traditional approaches typically strive to achieve the maximum statistical quality for the training set, believing that this will have the desired impact on the external validation set; in the present case, optimal descriptors computed from correlation weights aim to achieve the maximum statistical level for the calibration set, believing that this will increase statistical quality for the validation set.

Third, a pair of special indices (IIC and CII) [30,31] is used to characterize the observed correlations between experimental data and prediction.

The nature of these indices is not entirely understood. However, these indices can improve predictive potential. It can happen when acting individually [26,29]. It can occur when they act together [32]. In general, these indices has been tested quite effectively on various endpoints, such as, inhibitors against pancreatic cancer [33], the toxicity of dioxins [34], anti-colon activity [35], MCF-7 inhibitors [36], placental barrier permeability [37], toxicity of nitrobenzene derivatives to *Tetrahymena pyriformis* [38], anti-MES activity [39], binding affinity of endocrine disruptor [40], predicting the corneal permeability of drugs [41], modeling of antiproliferative inhibitors [42], development of potential therapeutics for pain treatment [43,44], QSAR studies of hepatitis C virus [45], in silico development of anesthetics [46].

The index of ideality of correlation [30] was conceived to ensure consistency between the correlation coefficient (determination coefficient) and the standard deviation. A common situation in model building is that a very high coefficient of determination is accompanied by a disproportionately large standard deviation or mean absolute error. This may occur because the regression equation on the training set is completely inappropriate for the validation set. It is also possible that the mean absolute error (mean square error) is quite acceptable, but because the values in the validation set are concentrated within a small interval, the coefficient of determination is small.

The correlation intensity index [31] is used to detect influential outliers that significantly reduce the correlation coefficient in a given set of molecular structures for a given endpoint. The impact of such outliers can be reduced by removing them from consideration. Another way to utilize this information about the state of the model on a given set of molecules is to attempt to change the method (replacing the set of molecular features extracted from SMILES for model construction). A set can be replaced by constructing a new list of attributes. This could be an extension of the list. For example, instead of correlation weighting of SMILES atoms (that is, parts of the SMILES string that cannot be considered separately), consider correlation weighting of linked pairs of SMILES atoms or even linked triplets of SMILES atoms. Another option is to switch from a model based on SMILES to one based on the invariants of a graph with hydrogen-atom vertices removed, or to a molecular graph that includes vertices representing hydrogen atoms. It is also possible to construct hybrid models using information extracted from both SMILES and the molecular graph. Such changes significantly alter the influence of the correlation intensity index. This means that the lists of potential outliers can change significantly from one method to another. However, there are cases where changing the method does not change the lists of potential outliers.

The combined influence of the correlation intensity index and the index of ideality of correlation on the Monte Carlo optimization process can be observed by changing the optimization process options. However, a simple interpretation of this impact on the optimization process is difficult because the influence of each index varies significantly. Table 2 confirms that applying the considered indices (TF_1_) improves the models’ predictive potential for NOAEL.

Table 3 shows the SMILES components that are associated with an increase or decrease in the NOAEL value for the target function TF_1_.

We can observe that the presence of sulfur (‘S’), branching (presence of brackets in SMILES), and cycles (denoted by digits) are factors in promoting increased NOAEL. Promoters of NOAEL decrease are the presence of oxygen atoms (‘O’), double bonds (‘=’), and nitrogen atoms (‘N’). These last elements are likely associated with higher molecular reactivity.

It is interesting to note that most SMILES attributes demonstrate both sign stability in their correlation weights (positive or negative across all Monte Carlo optimization runs) and relatively high frequencies of occurrence in the active, passive training sets, and in the calibration set. In principle, this is logical; pairs of SMILES atoms convey more information than a single SMILES atom. However, this circumstance complicates the interpretation of these results. The simplest part of the mechanistic interpretation of SMILES atom pairs is the increase in toxicity due to the influence of cycles, i.e., C…2 and c…3. However, markers of the location of double bonds (for example, O…= and C…=, which should be considered as promoters of decreased toxicity) are no less informative.

The range of the pNOAEL value on the set considered is (−3.8, 3.6). A collection of small molecules with more than 3 sulfur atoms is represented in Table 4.

One can see that all pNOAEL values in Table 4 are sufficiently large.

Comparison of NOAEL models from the literature with those obtained in this study confirms that the predictive potential of the models considered here is comparable to that of models reported in other studies (Table 5).

Table 5 shows that the previous models using CORAL software [16,17,22,23] have lower predictive potential than the new models obtained using the IIC, CII, and the Las Vegas algorithm. Results were quite modest in one case, with a validation R^2^ of 0.55 [16]. The previous versions of CORAL did not apply the IIC and CII components, and the training set was smaller. These factors are probably at the basis of the better results obtained with the present study.

## 5. Conclusions

The evaluation of the safety of substances often requires identifying an admissible intake dose. However, the number of experimental NOAEL values is quite limited, and the availability of further experimental values will be limited, due to the cost and time required for the experiment, and the use of animal studies, with the associated ethical and legal concerns (in vivo experiments are banned for cosmetics in Europe, for instance). For this reason, the possibility of having an in silico model is appealing. We developed new models that achieved good predictive performance on the external validation set across replicated splits. The described approach combines the Monte Carlo method and the Las Vegas algorithm to build models of repeated-dose toxicity. In addition, the index of ideality of correlation and the correlation intensity index are useful components of Monte Carlo optimization for calibrating a model on the calibration set. However, it should be noted that the time required to find solutions may be prohibitive, especially when considering large data sets. Furthermore, a stochastic process may yield the best solution, but it does not guarantee that the solution will be acceptable.

## Figures and Tables

**Table 1:** An example of a calculation optimal descriptor for O=C(O)C(Oc1ccc(cc1C)Cl)C.

DCW(3,15) = −2.8930 (sum of correlation weights)			
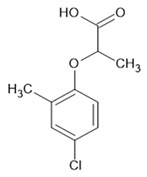			
**S_k_**	**CW(s_k_)**	**SS_k_**	**CW(SS_k_)**
O………	−0.1599		
=...........	0.1730	O…=…….	−0.4504
C………..	−0.7553	C…=…….	−0.0745
(………..	−0.7080	C…(…….	1.1275
O………..	−0.1599	O…(…….	−0.2548
(………..	−0.7080	O…(…….	−0.2548
C………..	−0.7553	C…(…….	1.1275
(………..	−0.7080	C…(…….	1.1275
O………..	−0.1599	O…(…….	−0.2548
c………..	−0.0266	c…O…….	−0.7650
1………..	−0.1888	c…1…….	0.2256
c………..	−0.0266	c…1…….	0.2256
c………..	−0.0266	c…c…….	−0.1935
c………..	−0.0266	c…c…….	−0.1935
(………..	−0.7080	c…(…….	0.2472
c………..	−0.0266	c…(…….	0.2472
c………..	−0.0266	c…c…….	−0.1935
1………..	−0.1888	c…1…….	0.2256
C………..	−0.7553	C…1…….	0.6797
(………..	−0.7080	C…(…….	1.1275
Cl……….	0.2129	Cl..(…….	0.3294
(………..	−0.7080	Cl..(…….	0.3294
C………..	−0.7553	C…(…….	1.1275

**Table 2:** The statistical characteristics of models for repeated-dose toxicity in rats were observed across five splits; the available data were divided into common training and validation sets.

TF	Split	Set *	n	R^2^	CCC	IIC	CII	Q^2^	RMSE	F	Na
TF_0_	1	C	210	0.5556	0.7446	0.7426	0.7801	0.5425	0.759	260	
		V	216	0.733	-	-	-	-	0.55	-	77
	2	C	210	0.3224	0.5659	0.4548	0.7263	0.2926	0.893	99	
		V	216	0.3787	-	-	-	-	0.91	-	74
	3	C	210	0.4081	0.6297	0.5581	0.7572	0.3886	0.893	143	
		V	216	0.7462	-	-	-	-	0.54	-	79
	4	C	210	0.4401	0.6372	0.5241	0.7715	0.4233	0.91	163	
		V	216	0.7545	-	-	-	-	0.70	-	77
	5	C	210	0.459	0.6713	0.6585	0.7367	0.4433	0.918	176	
		V	216	0.5537	-	-	-	-	0.85	-	74
TF_1_	1	C	210	0.719	0.8308	0.8479	0.832	0.7129	0.503	532	
		V	216	0.8221	-	-			0.48		77
		Vad	195	0.8391	-	-			0.48		
	2	C	210	0.734	0.8413	0.8567	0.8505	0.7289	0.404	574	
		V	216	0.7703	-	-	-	-	0.45	-	78
	3	C	210	0.7208	0.8291	0.849	0.8463	0.7162	0.405	537	
		V	216	0.7817	-	-	-	-	0.45	-	76
	4	C	210	0.6849	0.821	0.827	0.8349	0.6787	0.438	452	
		V	216	0.7351	-	-	-	-	0.47	-	74
	5	C	210	0.7357	0.8479	0.8576	0.855	0.7305	0.44	579	
		V	216	0.7212	-	-	-	-	0.48	-	70

^(^*^)^ C = calibration set; V = validation set; Vad = validation set for applicability domain; R^2^ = determination coefficient; CCC = concordance correlation coefficient; IIC = index of ideality of correlation [30]; CII = correlation intensity index [31]; Q^2^ = cross-validated R^2^; RMSE = root mean squared error; F = Fischer F-ratio; Na = the number of active (non-rare) SMILES attributes.

**Table 3:** Collection of promoters to increase or decrease the NOAEL.

Role	S_k_ & SS_k_	Run 1	Run 2	Run 3	NA	NP	NC	d_k_
Increase	C………..	1.3206	0.3511	0.8989	191	200	182	0.0002
	c………..	0.3771	0.1623	0.7608	118	112	160	0.0012
	S………..	2.9994	4.2808	2.6871	58	70	53	0.0008
	S…(…….	1.8022	2.9864	3.6560	41	50	33	0.0012
	S…C…….	1.5113	3.0866	2.9246	31	36	30	0.0005
	C…2…….	0.5991	1.1969	0.9958	23	22	26	0.0006
	c…3…….	0.4842	0.2804	0.1822	15	14	31	0.0027
	s………..	4.3465	4.2320	3.6971	13	6	9	0.0025
	s…c…….	4.0663	6.4475	4.3242	10	5	7	0.0022
	S…S…….	1.5115	3.3744	4.8729	8	7	9	0.0008
Decrease	O………..	−0.6029	−0.9352	−0.4391	154	145	150	0.0003
	=………..	−0.3701	−0.8934	−0.7882	126	137	139	0.0003
	O…=…….	−0.1676	−1.0169	−0.6973	115	114	112	0.0001
	C…=…….	−0.3762	−0.5888	−0.6866	104	117	108	0.0003
	O…(…….	−0.3247	−1.0059	−0.7540	89	103	102	0.0004
	O…C…….	−0.3595	−1.1412	−0.6789	81	74	74	0.0004
	(…(…….	−0.2227	−0.9002	−0.7885	70	59	81	0.0010
	N………..	−0.4003	−0.9350	−0.5850	64	63	110	0.0019
	=…(…….	−0.4170	−0.8823	−0.7449	54	71	74	0.0009
	c…O…….	−0.6217	−0.9549	−0.7509	20	21	37	0.0021

**Table 4:** A collection of compounds that contain sulfur atoms.

SMILES	pNOAEL Experimental
CC1SSC(C)SS1	2.965
C=CCSSSSC	2.900
CSSSSC	2.834
C1SSCSSS1	2.974
CCCSSSSC	2.905
o1ccc(c1C)SSSSc2ccoc2C	2.715

**Table 5:** Comparison of predictive potentials of NOAEL models.

n Validation	R^2^ Validation	RMSE Validation	Reference
27	0.94	0.14	[15]
141	0.55	0.45	[16]
38	0.82	0.57	[17]
79	0.71	0.59	[22]
22	0.67	0.36	[23]
-	-	0.53	[24]
25	0.87	0.59	[25]
216	0.76	0.47	In this study

## Data Availability

The original contributions presented in this study are included in the article/Supplementary Materials. Further inquiries can be directed to the corresponding author.

## Supplementary Materials

The following supporting information can be downloaded at: https://www.mdpi.com/article/10.3390/toxics14040338/s1, Table S1: contains data on the percentage of matching distributions for the five splits considered for TF_1_. Table S2: Technical details for the split 1 (TF_1_). Table S3: Full statistics on all the training active and passive splits for TF_1_.

## References

[1] Majid S., Reus A., Hoondert R., Blokker M., Dash A., Houtman C., Schriks M., Dingemans M.M.L. (2025). A framework for evaluating less-than-lifetime exposures: Advancing toxicological risk assessment for drinking water quality. Arch. Toxicol..

[2] Kimmel C.A. (2001). U.S. EPA reference dose/reference concentration methodology: Update on a review of the process. Hum. Ecol. Risk Assess. Int. J..

[3] (2025). Repeated Dose 28-Day Oral Toxicity Study in Rodents, OECD Guidelines for the Testing of Chemicals, Section 4.

[4] (1998). Repeated Dose 90-Day Oral Toxicity Study in Non-Rodents, OECD Guidelines for the Testing of Chemicals, Section 4.

[5] (2018). Chronic Toxicity Studies, OECD Guidelines for the Testing of Chemicals, Section 4.

[6] (2025). Combined Repeated Dose Toxicity Study with the Reproduction/Developmental Toxicity Screening Test, OECD Guidelines for the Testing of Chemicals, Section 4.

[7] Dorne J.L.C.M., Fink-Gremmels J. (2013). Human and animal health risk assessments of chemicals in the food chain: Comparative aspects and future perspectives. Toxicol. Appl. Pharmacol..

[8] EU—Communication from the Commission to the European Parliament, the Council, the European Economic and Social Committee and the Committee of the Regions. Chemicals Strategy for Sustainability: Towards a Toxic-Free Environment. COM, 2020, 667 Final. https://eur-lex.europa.eu/legal-content/EN/TXT/HTML/?uri=CELEX:52020DC0667.

[9] Herzler M., Marx-Stoelting P., Pirow R., Riebeling C., Luch A., Tralau T., Schwerdtle T., Hensel A. (2021). The “EU chemical strategy for sustainability” questions regulatory toxicology as we know it: Is it all rooted in sound scientific evidence?. Arch. Toxicol..

[10] Barile F.A., Berry S.C., Blaauboer B., Boobis A., Bolt H.M., Borgert C., Dekant W., Dietrich D., Domingo J.L., Galli C.L. (2021). The EU chemicals strategy for sustainability: In support of the BfR position. Arch. Toxicol..

[11] Sewell F., Corvaro M., Andrus A., Burke J., Daston G., Delaney B., Domoradzki J., Forlini C., Green M.L., Hofmann T. (2022). Recommendations on dose level selection for repeat dose toxicity studies. Arch. Toxicol..

[12] ECHA—European Chemical Agency (2021). Critical Aspects for Designing and Conducting Extended One-Generation Reproductive Toxicity (EOGRT) Studies Under REACH.

[13] ECHA—European Chemical Agency (2022). Advice on Dose-Level Selection for the Conduct of Reproductive Toxicity Studies (OECD TGs 414, 421/422 and 443) Under REACH.

[14] ECETOC—European Centre for Ecotoxicology and Toxicology of Chemicals (ECETOC) Guidance on Dose Selection. Technical report No. 138. Brussels, March 2021. ISSN-2079-1526-138 (Online). https://www.ecetoc.org/wp-content/uploads/2021/10/ECETOC-TR-138-Guidance-on-Dose-Selection_Final.pdf.

[15] Ghosh S., Roy K. (2024). Quantitative read-across structure-activity relationship (q-RASAR): A novel approach to estimate the sub chronic oral safety (NOAEL) of diverse organic chemicals in rats. Toxicology.

[16] Selvestrel G., Lavado G.J., Toropova A.P., Toropov A.A., Gadaleta D., Marzo M., Baderna D., Benfenati E. (2022). Monte Carlo models for sub-chronic repeated-dose toxicity: Systemic and organ-specific toxicity. Int. J. Mol. Sci..

[17] Gadaleta D., Marzo M., Toropov A.A., Toropova A.P., Lavado G.J., Escher S.E., Dorne J.L.C.M., Benfenati E. (2021). Integrated In silico models for the prediction of No-Observed-(Adverse)-Effect Levels and Lowest-Observed-(Adverse)-Effect Levels in rats for sub-chronic repeated-dose toxicity. Chem. Res. Toxicol..

[18] HESS Database, Available from the OECD QSAR Toolbox. https://qsartoolbox.org/.

[19] Watford S., Pham L.L., Wignall J., Shin R., Martin M.T., Friedman K.P. (2019). ToxRefDB version 2.0: Improved utility for predictive and retrospective toxicology analyses. Reprod. Toxicol..

[20] Carnesecchi E., Mostrag A., Ciacci A., Roncaglioni A., Tarkhov A., Gibin D., Sartori L., Benfenati E., Yang C., Dorne J.L.C.M. (2023). OpenFoodTox: EFSA’s Chemical Hazards Database (Versione 6) [Data Set].

[21] Prussia A.J., Welsh C., Somers T.S., Ruiz P. (2024). Workflow for predictive risk assessments of UVCBs: Cheminformatics library design, QSAR, and read-across approaches applied to complex mixtures of metal naphthenates. Front. Toxicol..

[22] Toropova A.P., Toropov A.A., Marzo M., Escher S.E., Dorne J.L., Georgiadis N., Benfenati E. (2018). The application of new HARD-descriptor available from the CORAL software to building up NOAEL models. Food Chem. Toxicol..

[23] Toropova A.P., Toropov A.A., Veselinović J.B., Veselinović A.M. (2015). QSAR as a random event: A case of NOAEL. Environ. Sci. Pollut. Res..

[24] Hisaki T., Aiba Née Kaneko M., Yamaguchi M., Sasa H., Kouzuki H. (2015). Development of QSAR models using artificial neural network analysis for risk assessment of repeated-dose, reproductive, and developmental toxicities of cosmetic ingredients. J. Toxicol. Sci..

[25] Vázquez-Valadez V.H., Oliva-Arellano M.V., Martínez-Soriano P.A., Hernández-Serda M.A., Velázquez-Sánchez A.M., Concepción Rodríguez-Maciel J., Ángeles E. (2023). In Silico Predictability of Toxicity Parameters Using the OECD QSAR Toolbox of Some Components of Cannabis sativa. ChemistrySelect.

[26] Makari Z., Shiri F., Ahmadi S. (2025). Computational prediction of sublimation enthalpy of organic compounds using the Monte Carlo technique. Chem. Phys..

[27] Yang C., Rathman J.F., Magdziarz T., Mostrag A., Kulkarni S., Barton-Maclaren T.S. (2021). Do similar structures have similar no observed adverse effect level (NOAEL) values? Exploring cheminformatics approaches for estimating NOAEL bounds and uncertainties. Chem. Res. Toxicol..

[28] Cronin M.T.D., Enoch S.J., Mellor C.L., Przybylak K.R., Richarz A.N., Madden J.C. (2017). In silico prediction of organ level toxicity: Linking chemistry to adverse effects. Toxicol. Res..

[29] Veselinović A.M., Toropova A.P., Toropov A.A., Roncaglioni A., Benfenati E. (2025). Las Vegas algorithm in the prediction of intrinsic solubility of drug-like compounds. J. Mol. Graph. Model..

[30] Toropov A.A., Toropova A.P. (2017). The index of ideality of correlation: A criterion of predictive potential of QSPR/QSAR models?. Mutat. Res.-Genet. Toxicol. Environ. Mutagen..

[31] Toropov A.A., Toropova A.P. (2020). Correlation intensity index: Building up models for mutagenicity of silver nanoparticles. Sci. Total Environ..

[32] Bhawna B., Kumar S., Kumar P., Kumar A. (2024). Correlation intensity index-index of ideality of correlation: A hyphenated target function for furtherance of MAO-B inhibitory activity assessment. Comput. Biol. Chem..

[33] Ahmadi S., Lotfi S., Azimi A., Kumar P. (2024). Multicellular target QSAR models for predicting of novel inhibitors against pancreatic cancer by Monte Carlo approach. Results Chem..

[34] Kumar P., Kumar A., Singh D. (2022). CORAL: Development of a hybrid descriptor based QSTR model to predict the toxicity of dioxins and dioxin-like compounds with correlation intensity index and consensus modelling. Environ. Toxicol. Pharmacol..

[35] Askarzade A., Ahmadi S., Almasirad A. (2025). SMILES-based QSAR and molecular docking studies of chalcone analogues as potential anti-colon cancer. Sci. Rep..

[36] Valizadeh N., Ahmadi S., Lotfi S., Ketabi S. (2025). Integrating QSAR modeling, ADMET screening, molecular docking, and molecular dynamics simulations to identify potential MCF-7 inhibitors. Comput. Biol. Med..

[37] Vukomanović P., Stefanović M., Stevanović J.M., Petrić A., Trenkić M., Andrejević L., Lazarević M., Sokolović D., Veselinović A.M. (2024). Monte Carlo optimization method based QSAR modeling of placental barrier permeability. Pharm. Res..

[38] Ouabane M., Zaki K., Tabti K., Alaqarbeh M., Sbai A., Sekkate C., Bouachrine M., Lakhlifi T. (2024). Molecular toxicity of nitrobenzene derivatives to tetrahymena pyriformis based on SMILES descriptors using Monte Carlo, docking, and MD simulations. Comput. Biol. Med..

[39] Zivadinovic B., Stamenović J., Živadinović J., Živadinović L., Živadinović A., Stojanović M., Lazarevic M., Sokolović D., Veselinović A.M. (2023). Monte Carlo optimization based QSAR modeling, molecular docking studies, and ADMET predictions of compounds with anti-MES activity. Struct. Chem..

[40] Goyal S., Rani P., Chahar M., Hussain K., Kumar P., Sindhu J. (2023). Quantitative structure activity relationship studies of androgen receptor binding affinity of endocrine disruptor chemicals with index of ideality of correlation, their molecular docking, molecular dynamics and ADME studies. J. Biomol. Struct. Dyn..

[41] Živković M., Zlatanović M., Zlatanovic N., Golubović M., Veselinović A.M. (2022). A QSAR model for predicting the corneal permeability of drugs—The application of the Monte Carlo optimization method. New J. Chem..

[42] Tabti K., El Mchichi L., Sbai A., Maghat H., Bouachrine M., Lakhlifi T. (2022). Molecular modelling of antiproliferative inhibitors based on SMILES descriptors using Monte-Carlo method, docking, MD simulations and ADME/Tox studies. Mol. Simul..

[43] Perić V., Golubović M., Lazarevic M., Marjanović V., Kostić T., Đorđević M., Milić D., Veselinović A.M. (2021). Development of potential therapeutics for pain treatment by inducing Sigma 1 receptor antagonism: In silico approach. New J. Chem..

[44] Golubović M., Kostić T., Djordjević M., Perić V., Lazarevic M., Milić D., Marjanović V., Veselinović A.M. (2021). In silico development of potential therapeutic for the pain treatment by inhibiting voltage-gated sodium channel 1.7. Comput. Biol. Med..

[45] Ghiasi T., Ahmadi S., Ahmadi E., Talei Bavil Olyai M.R., Khodadadi Z. (2021). The index of ideality of correlation: QSAR studies of hepatitis C virus NS3/4A protease inhibitors using SMILES descriptors. SAR QSAR Environ. Res..

[46] Stošić B., Janković R., Stošic M., Markovic D., Stanković D., Sokolović D., Veselinović A.M. (2020). In silico development of anesthetics based on barbiturate and thiobarbiturate inhibition of GABAA. Comput. Biol. Chem..

